# Neuroimaging in the pre-ictal or premonitory phase of migraine: a narrative review

**DOI:** 10.1186/s10194-023-01617-x

**Published:** 2023-08-11

**Authors:** Nazia Karsan, Peter J. Goadsby

**Affiliations:** 1 Headache Group, NIHR King’s Clinical Research Facility and SLaM Biomedical Research Centre, The Wolfson Sensory, Pain and Regeneration Research Centre, Institute of Psychiatry, Psychology and Neuroscience, King’s College London, London, SE5 9PJ UK; 2 Department of Neurology, University of California, Los Angeles, USA

**Keywords:** Migraine, Premonitory, Prodrome, Neuroimaging, fMRI, ASL, PET, DTI, Hypothalamus

## Abstract

**Background:**

The premonitory phase, or prodrome, of migraine, provides valuable opportunities to study attack initiation and for treating the attack before headache starts. Much that has been learned about this phase in recent times has come from the outcomes of functional imaging studies. This review will summarise these studies to date and use their results to provide some feasible insights into migraine neurobiology.

**Main body:**

The ability to scan repeatedly a patient without radiation and with non-invasive imaging modalities, as well as the recognition that human experimental migraine provocation compounds, such as nitroglycerin (NTG) and pituitary adenylate cyclase activating polypeptide (PACAP), can trigger typical premonitory symptoms (PS) and migraine-like headache in patients with migraine, have allowed feasible and reproducible imaging of the premonitory phase using NTG. Some studies have used serial scanning of patients with migraine to image the migraine cycle, including the ‘pre-ictal’ phase, defined by timing to headache onset rather than symptom phenotype. Direct observation and functional neuroimaging of triggered PS have also revealed compatible neural substrates for PS in the absence of headache. Various imaging methods including resting state functional MRI (rsfMRI), arterial spin labelling (ASL), positron emission tomography (PET) and diffusion tensor imaging (DTI) have been used. The results of imaging the spontaneous and triggered premonitory phase have been largely consistent and support a theory of central migraine attack initiation involving brain areas such as the hypothalamus, midbrain and limbic system. Early dysfunctional pain, sensory, limbic and homeostatic processing via monoaminergic and peptidergic neurotransmission likely manifests in the heterogeneous PS phenotype.

**Conclusion:**

Advances in human migraine research, including the use of functional imaging techniques lacking radiation or radio-isotope exposure, have led to an exciting opportunity to study the premonitory phase using repeated measures imaging designs. These studies have provided novel insights into attack initiation, migraine neurochemistry and therapeutic targets. Emerging migraine-specific therapies, such as those targeting calcitonin gene-related peptide (CGRP), are showing promise acutely when taken during premonitory phase to reduce symptoms and prevent subsequent headache. Therapeutic research in this area using PS for headache onset prediction and early treatment is likely to grow in the future.

## Background

Migraine is the most common of the primary headache disorders and affects people at the peak of their working lives, thereby causing significant socio-economic burden [1]. Whilst initially thought of as a disorder of headache, the migraine phenotype extends beyond headache, and with widespread brain dysfunction involving pain, sensory, homeostatic and limbic systems [2]. The symptoms associated with migraine are therefore heterogeneous and can include mood and cognitive dysfunction, altered sleep, arousal and feeding [3], as well as headache.

Some symptoms can start before headache onset, and in some patients can reliably warn of impending headache [4]. These have been termed premonitory symptoms (PS), or erroneously mislabelled [5] as the prodrome in the current iteration of the International Classification of Headache Disorders, third edition (ICHD-3) [6]. PS occur before headache and in its absence and understanding the biological mediation of PS and their neuroanatomical substrates could provide insights into migraine attack initiation. PS include sensory sensitivities, such as photic hypersensitivity, phonophobia, movement sensitivity, arousal changes with sleep cycle changes or extreme fatigue and difficulty staying awake, cognitive changes such as word finding difficulty, difficulty with reading or writing and concentration change and neuroendocrine symptoms like thirst, polyuria, food cravings and yawning [3].

PS also provide a therapeutic window for early attack treatment in patients who can reliably identify them for the purposes of headache prediction. There have been some encouraging results in small historical studies using domperidone, a dopamine antagonist versus placebo [7, 8], and in an open label study of naratriptan, a 5HT_1B/1D_ receptor agonist [9], when taken during the premonitory phase. The drugs in this class, termed the *triptans*, were the first migraine specific drugs developed in the 1990’s [10] and there are now seven available. Some of the triptan studies in the literature suggest that headache is needed for therapeutic efficacy and that they are not effective when taken early, such as during migraine aura in those who get these symptoms prior to headache [11, 12]. One open label study with oral sumatriptan suggested it was useful in aura [13], although given the pharmacokinetics of the drug [14], the data are not compelling and larger, placebo-controlled, randomised studies using parenteral formulations are warranted.

More recently, novel targeted treatments acting on the CGRP pathway have been developed and are changing the landscape of migraine treatment by providing well tolerated and specific targeted treatments for the first time since the triptan era. These have been shown in early studies to be effective acutely at preventing headache when taken during PS (the small molecule CGRP receptor antagonist, ubrogepant) [15], and may be effective in reducing other symptoms and disability when taken in the premonitory phase [16], and preventively CGRP-targeted therapies may also have an effect on non-headache symptoms such as cognitive dysfunction (a CGRP ligand-binding monoclonal antibody, fremanezumab) [17], as well as on the occurrence of PS (a CGRP ligand-binding monoclonal antibody, galcanezumab) [18]. These results provide hope for an exciting future in migraine therapeutics. Despite a wealth of pathophysiological and therapeutic research in migraine, there has never been an unambiguously effective treatment for all those afflicted, and headache prediction and early treatment has not been a widely used strategy before. Clearly for both patients and physicians alike, the ability to prevent headache through early attack treatment is an attractive strategy for limiting attack-related disability and attack duration.

Studying the biological mediation of PS and the systems involved in attack initiation are therefore important, both to advance migraine pathophysiological understanding, and for future therapeutics development. One of the means that has been used in recent times has been the increasing use of functional imaging methodologies to study migraine brain structure and function. Capturing spontaneous migraine attacks using these study designs has inherent challenges, which are particularly pronounced when it comes to imaging PS. These symptoms may not necessarily be promptly associated with migraine by patients, and in some, the time to headache onset is short [4], making them logistically difficult to capture reliably within imaging study designs. Some study designs have therefore used the ability to image a patient with migraine serially, that is repeatedly on consecutive days, or serially in the interictal state, to capture the entirety of the migraine cycle, including the lead up to headache, or the ‘pre-ictal’ phase [19–27]. Whilst these studies have not systematically studied the clinical phenotype of the patient during this time *per se* and have used the time to headache onset to define the ‘pre-ictal’ phase, the demonstration of dynamic and oscillating changes within the migraine brain throughout the pre-ictal, ictal, post-ictal and interictal phases of the migraine cycle have been exciting advances to the understanding of the ‘state and trait’ model of migraine. There are felt to be likely underlying alterations in sensory processing and brainstem function, which are present interictally, and further changes in the lead up to and during a migraine attack, may account for the interictal sensory sensitivities and other symptoms that some patients experience, as well as PS and attack-related symptoms.

Due to the difficulties in examining spontaneous attacks, some studies have used other methods to image the pre-ictal or pre-headache phase of migraine. Some of these have included the assessment of brain responses of patients with migraine compared to healthy controls to nociceptive or olfactory stimulation of the trigeminal system at intervals using fMRI [27, 28]. Others have used the imaging of triggered PS [29–35]. Whilst human experimental migraine models have been used in migraine research for some time [36, 37], it has only been through the increasing recognition of the importance of PS in migraine neurobiology and treatment, that led to the discovery that some of these compounds could trigger PS as well as migraine-like headache in susceptible individuals [38–41]. Namely, NTG, a vasodilator which provokes migraine via nitric oxide mechanisms [38, 39, 41] and PACAP, a vasodilatory and inflammatory neuropeptide which provokes migraine via the PAC1 or VPAC receptors [40], are the ones that seem to most commonly provoke PS, whereas CGRP less so [40], though it has a high affinity for provoking migraine-like headache in patients with migraine [42]. The NTG model has been exploited with imaging, and has provided an exciting opportunity to repeatedly image PS in a reliable fashion and has helped to provide supportive evidence for early neural dysfunction in migraine.

This review will summarise the current up-to-date imaging studies of the pre-ictal: for the purpose of this review, defined as the pre-headache phase of migraine irrespective of clinical symptoms, or premonitory phase: the symptomatic phase preceding headache of migraine. We will then discuss the insights that the results of these offer into migraine biology and novel therapeutics development.

### Pre-ictal imaging

#### Imaging the migraine cycle to capture the pre-ictal phase

Historical imaging studies used the administration of inhaled [43] or intra-arterial [44] Xenon133 to measure cerebral blood flow (CBF) during migraine. CBF is a commonly used surrogate for neuronal activity, based on the concept of neurovascular coupling. There was a suggestion of occipitoparietal reduced CBF preceding migraine headache in migraine with aura in these studies, which spread anteriorly, and headache occurred during this oligaemia [44]. These findings were not reproduced using these models in migraine without aura [45], so these findings were assumed to be imaging correlates of cortical spreading depression (CSD), the neurophysiological phenomenon of cortical depolarisation followed by repolarisation, which is thought to mediate aura symptoms in humans.

A subsequent study by Stankewitz and colleagues in 2011 used a previously validated model of trigeminal nociception along with behavioural assessment and fMRI [46], to study the migraine cycle in patients with migraine and compare this to healthy controls during the interictal, pre-ictal and ictal phases [27]. Using a combination of these results, the authors found that the level of trigeminal activation on fMRI in response to nociceptive stimulation increases over the migraine cycle and peaks ictally, and the next migraine attack can be predicted by signal intensity in the spinal nucleus. Dorsolateral pontine activation seemed to occur later, and this had previously been demonstrated during spontaneous [47–49] and triggered [50] migraine headache. This study for the first time, suggested that oscillating responses to nociceptive stimulation in migraine occur, and implicated areas of possible attack generation outside of the pontine, midbrain and hypothalamic areas that had been demonstrated on imaging studies during migraine headache before [47–52].

Further studies followed using the approach of imaging the migraine cycle with trigeminal nociceptive stimulation, aiming to capture imaging responses in the lead up to migraine headache and the pre-ictal phase. Schulte and colleagues imaged a patient with migraine every day for 30 days and managed to image 3 spontaneous attacks during this time [20]. This study demonstrated increased hypothalamic activity on nociceptive triggered blood oxygen level dependant (BOLD) fMRI in the lead up to headache, and also altered functional connectivity between the hypothalamus and the spinal trigeminal nuclei and dorsolateral pons on the day before migraine headache, providing supportive evidence for dynamic changes in hypothalamic and brainstem connectivity throughout the migraine cycle, with early changes preceding headache. A follow-up study used the same protocol in 9 subjects with migraine with daily imaging for 30 days, and acquired data from 7 subjects and 27 spontaneous attacks [19]. The authors found corroborative evidence for hypothalamic activation on BOLD fMRI in response to trigeminal nociceptive stimulation in the 48 h prior to migraine headache and suggested that this interval should be used to define the pre-ictal or premonitory phase of migraine on future imaging studies. Resting state functional connectivity analyses, in 8 of the 9 subjects demonstrated increased connectivity between the right nucleus accumbens and the left amygdala, hippocampus and parahippocampal gyrus in the pre-ictal phase, as well as increased connectivity between this region and the dorsorostral pons [25]. As well as the hypothalamus, this study demonstrated the likely involvement of other limbic and dopaminergic brain areas preceding headache in migraine.

Other groups have used different approaches to image the phase preceding headache without necessarily examining clinical symptoms. One study scanned the different phases of migraine in patients with migraine, by performing periodic interictal scans, and thereafter identifying that some scans were conducted in the 72 h ahead of headache, and others in the 24 h preceding headache. The authors used a range of fMRI methods, including infra-slow oscillation power, regional homogeneity and functional connectivity analyses, and found increased infra-slow oscillatory activity in the hypothalamus and brainstem in the 24 h preceding migraine headache, and increased midbrain and hypothalamus functional connectivity and regional homogeneity during this time. These findings were not present in the post-ictal phase following migraine headache, and interictally and were significantly different to healthy controls. Using different fMRI methods, the authors provided additional supportive evidence for altered hypothalamic and brainstem function in the lead up to migraine headache by conducting serial fMRI imaging in 31 patients with migraine and 31 healthy controls, therefore again capturing the pre-headache period and used noxious orofacial stimulation and resting state functional connectivity analyses, specifically in the brainstem [24]. Sensitivity to pain was found to be heightened interictally, with a decrease immediately before migraine and increased activation of the spinal trigeminal nucleus in response to stimulation was present pre-ictally. There was reduced functional connectivity between the spinal trigeminal nucleus and the rostroventral medulla during this time. Interictally, different changes occurred between the midbrain and rostroventral medulla. The authors postulated the pre-ictal enhancement of endogenous nociceptive mechanisms and that cyclical fluctuations in brainstem activity occur throughout the migraine cycle. This study again supported the theory that deep brain structures are involved in the lead up to a migraine headache, and also that pain sensitivity thresholds are altered throughout the migraine cycle [53], suggesting reduced sensory habituation and inhibitory mechanisms pre-ictally.

Diffusion tensor imaging (DTI) was then used to examine microstructural brainstem changes pre-ictally in migraine [54]. Interictal mean diffusivity changes in the midbrain, dorsomedial pons and spinal trigeminal nucleus normalised to control levels in the pre-ictal period 24 h ahead of headache and were then altered again for 72 h thereafter. These regional brainstem anatomical changes are therefore postulated to fluctuate over the migraine cycle, in particular pre-ictally, when they may be involved in activation or sensitisation of ascending trigeminal nociceptive pathways. A follow-up study aimed to investigate the relationship between hypothalamic and brainstem activation pre-ictally further, using a combination of CBF (using pseudocontinuous arterial spin labelling, pCASL) and functional connectivity measurements [22]. The authors found reduced blood flow in the lateral hypothalamus in the 24 h leading up to headache and reduced hypothalamic functional connectivity with brainstem regions such as the periacqueductal gray (PAG), dorsal pons, rostral ventromedial medulla, as well as in cingulate cortex. This study suggested that the hypothalamus is involved in attack initiation and is involved in brainstem pain processing sensitivity through connectivity changes before headache, thereby facilitating migraine headache via either internal or external triggering mechanisms. A final study by these authors examined these changes in more detail by imaging a cohort of patients with migraine and healthy controls 5 days a week for 4 weeks, and demonstrated that resting brainstem activity varied most dramatically in the 24 h preceding headache in the migraine group in the regions of the dorsal pons and spinal trigeminal nucleus [21]. Interestingly, the authors report that no subjects reported prodromal symptoms in the 24 h preceding headache, suggesting that they were asymptomatic during those scans. This study once again supported the dynamic changes in brainstem circuitry during the migraine cycle and together these studies confirm the role of the hypothalamus and its connections with trigeminal pain processing brainstem structures in the lead up to migraine headache.

A recent fMRI study looked at intrinsic network connectivity during spontaneous migraine attacks and follow-up imaging ahead of the next attack [26]. This study suggested involvement of networks outside of hypothalamic-brainstem ones, and implicated cyclical changes in visual, auditory, somatosensory and limbic networks, which increase over the migraine cycle and peak pre-ictally and return to baseline during headache. A change in network integrity across sensory and limbic networks could feasibly be involved given the symptom phenotype that patients experience in the lead up to headache. The interaction between these pathways and those involving the hypothalamus and brainstem structurally and functionally are therefore of interest, and the top-down modulation of trigeminal sensory processing from the pre-ictal phase through to migraine headache may have a role via such pathways in thresholding ensuing headache, either endogenously or following exposure to an external migraine trigger.

The imaging studies from 2011 onwards imaging the pre-ictal phase of migraine are summarised in Table 1.

**Table 1:** Summary of the currently published pre-ictal imaging studies using serial imaging in migraine

Study	Imaging modality	Subject cohort	Study design	Main findings
Stankewitz *et al*., 2011 [27]	fMRI with nociceptive stimulation (gaseous ammonia as nociceptive stimulus)	Migraine (*n* = 20) Healthy controls (*n* = 20)	Interictal imaging at least 72 h before next and 72 h following last migraine attack (additional 10 subjects pre-ictal, 13 ictal on post hoc analyses)	Trigeminal activation peaks ictally The next migraine attack can be predicted by signal intensity in the spinal nucleus
Schulte *et al*., 2016 [20]	Task-evoked fMRI with nociceptive stimulation (visual stimulation, gaseous ammonia nociceptive stimulation, rose odour olfactory stimulation and air control)	One migraine without aura subject	Daily scanning for 30 days, capturing 3 spontaneous attacks	Increased hypothalamic activity in the lead up to headache Altered functional connectivity between hypothalamus and spinal trigeminal nuclei and dorsolateral pons the day before headache
Meylakh* et al*., 2018 [23]	Resting state fMRI with infra-slow oscillatory activity, regional homogeneity, and connectivity analyses	Migraine (*n* = 26) Healthy controls (*n* = 78)	Serial ‘interictal’ scans, capturing 11 subjects 72 h and 8 subjects 24 h before headache, 26 interictal	Increased infra-slow oscillatory activity in hypothalamus and brainstem 24 h preceding migraine headache. Increased midbrain and hypothalamus functional connectivity and regional homogeneity 24 h preceding headache
Marciszewski *et al.,* 2018 [24]	fMRI with nociceptive stimulation (specifically brainstem)	Migraine (*n* = 31) Healthy controls (*n* = 60)	Serial ‘interictal’ scans, capturing 10 subjects 72 h and 10 subjects 24 h before headache, 28 interictal	Increased activation of spinal trigeminal nucleus pre-ictally Reduced functional connectivity between spinal trigeminal nucleus and rostroventral medulla pre-ictally
Marciszewski *et al*., 2019 [54]	DTI	Migraine (*n* = 36) Healthy controls (*n* = 29)	Serial ‘interictal’ scans, capturing 15 subjects 72 h and 13 subjects 24 h before headache, 31 interictal	Interictal mean diffusivity changes in midbrain, dorsomedial pons and spinal trigeminal nucleus, normalised to control levels 24 h ahead of headache, and were then altered again for 72 h thereafter
Meylakh* et al*., 2020 [22]	Resting state fMRI with pCASL and connectivity analysis	Migraine (*n* = 24) Healthy controls (*n* = 26)	Serial ‘interictal’ scans, capturing 13 subjects 72 h after headache and 7 subjects 24 h before headache, 22 interictal	Reduced blood flow in lateral hypothalamus 24 h leading up to headache Reduced hypothalamic functional connectivity with PAG, dorsal pons, rostral ventromedial medulla and cingulate cortex
Schulte *et al*., 2020 [19]	Task-evoked fMRI with nociceptive stimulation (visual stimulation, gaseous ammonia nociceptive stimulation, rose odour olfactory stimulation and air control)	Migraine (*n* = 7)	Daily scanning for 30 days, capturing 27 attacks	Hypothalamic activation in the 48 h prior to headache
Schulte *et al*., 2020 [25]	Resting state fMRI with connectivity analysis	Migraine (*n* = 8)	Daily scanning for 30 days, capturing 15 attacks	Increased connectivity between right nucleus accumbens and left amygdala, hippocampus and parahippocampal gyrus in the pre-ictal phase Increased connectivity between nucleus accumbens and dorsorostral pons
Meylakh* et al*., 2021 [21]	Resting state fMRI	Migraine (*n* = 3) Healthy controls (*n* = 5)	Scanning 5 days a week for 4 weeks, acquiring interictal and pre-ictal scans within 72 or 24 h of headache	Resting brainstem activity most variable 24 h preceding headache, in dorsal pons and spinal trigeminal nucleus
Stankewitz *et al*., 2022 [26]	Resting state fMRI with network connectivity analysis	Migraine (*n* = 12)	Serial ‘interictal’ scans	Cyclical changes in visual, auditory, somatosensory and limbic networks increasing over migraine cycle and peaking pre-ictally and returning to baseline during headache

#### Triggering the migraine attack to capture the pre-ictal phase

Given the challenges in capturing the pre-ictal phase of migraine spontaneously, and the need for serial imaging of spontaneous attacks to able to do this, the imaging of pharmacologically triggered attacks using human experimental migraine models has been used by some groups. One such study examined the response of the blood oxygen-level dependant level (BOLD) contrast on fMRI in the region of the hypothalamus at baseline and in response to an oral glucose challenge in both spontaneous (*n* = 5) and NTG-triggered migraine (*n* = 16) during the premonitory phase [34], and in healthy controls (*n* = 11). All migraine patients and healthy controls were imaged at baseline before any drug administration on one visit and remained in the scanner for further imaging following an oral glucose challenge after around 7 minutes. NTG-triggered patients and healthy controls were imaged following oral glucose ingestion at a fixed 90-min interval following NTG administration irrespective of clinical symptoms, but with the hope of imaging during PS in the ones with migraine on a different visit. PS were reported following NTG infusion at 90 min by 13/16 subjects, but these were not compared to patient’s spontaneous symptoms to assess if they were typical. Spontaneous attack patients were asked to present for imaging as soon as they felt any symptoms of a migraine, but the authors do not elaborate on the premonitory phenotype in these subjects. The results suggested altered hypothalamic neuronal function in the pre-ictal phase of migraine only, with a steeper and faster recovery in BOLD signal in the hypothalamus following glucose administration in both spontaneous and NTG-triggered attacks during the premonitory phase compared to healthy controls.

The same group used MR spectroscopy to image NTG-triggered attacks at 3 intervals (baseline ahead of any drug exposure, following NTG exposure before migraine headache irrespective of symptoms and during NTG-provoked migraine-like headache) and measured glutamate, glutamine and gamma-aminobutyric acid (GABA) over a volume of interest in the visual cortex [55]. Whilst glutamate and glutamine concentrations did not change over the migraine cycle, GABA increased from the interictal to pre-ictal states. This study suggested that increased GABA levels may be involved in the brain’s response in trying to inhibit sensory hypersensitivity or pain in the pre-ictal phase.

These triggered studies have again provided evidence for altered hypothalamic activity before migraine headache in both spontaneous and triggered attacks, and have supported the idea that triggered attacks are a reliable way to study PS. There may be an increase in sensory inhibition via GABA before migraine headache. This may be involved in reducing pain sensitivity in the pre-ictal phase and in mediating some of the oscillating changes in brainstem pain processing structures function that have been demonstrated on the various imaging studies. The changes in sensory thresholding before migraine headache may alter the occurrence of headache thereafter via neurotransmitter alterations, depending on internal or external trigger exposure. We have previously shown that following NTG exposure, some patients with migraine can develop typical PS for them (similar to spontaneous attacks), but these are not necessarily followed by delayed migraine-like headache [39]. This concept of typical PS being present or being provoked but not being followed by migraine headache is interesting, as it provides a possible clinical correlate for such a sensory thresholding effect. Further systematic prospective diary studies of PS and possible triggers in spontaneous attacks would be needed to assess this in more detail.

### Premonitory imaging

The first study that imaged the symptomatic premonitory phase of migraine was conducted by Maniyar and colleagues in 2014, and used H_2_^15^0 PET imaging in NTG-provoked attacks [30]. This imaging methodology uses labelled water as an administered radioisotope, which acts as a freely diffusible tracer that enters cerebral tissue without being metabolised. CBF changes can be measured following equilibrium, and arterial blood sampling via an arterial line is conducted. Eight subjects with migraine were scanned at baseline, during the symptomatic premonitory phase and during migraine-like headache. Increases in blood flow were found in areas such as the hypothalamus, midbrain, dorsal pons and cortical areas such as prefrontal and occipital cortex. This study for the first time, provided functional association between brain changes and the clinical phenotype that patients experienced during the imaging. Further studies by the same authors tried to characterise which changes could be responsible for particular symptoms (neural substrates for specific PS), and identified that occipital activation in extrastriate visual cortex was increased in those with premonitory photic hypersensitivity compared to those without this symptom [29], and similarly that activation in the region of the rostral dorsal medulla and PAG (the brain’s emesis network) was only present in those with premonitory nausea and not in those without [31]. These studies provided feasible neural correlates for typical PS and linked the brain findings to the clinical symptoms that patients experience. Pre-clinical work has suggested the role of hypothalamic [56–59], midbrain [60–62], medullary [63–65] and pontine [64, 66] areas in trigeminal nociceptive processing, providing possible links between PS and headache, and also feasible links between physiological symptoms such as those regulating sleep, mood and feeding with PS and migraine in general, and imaging studies have supported this findings.

Further studies have followed using different imaging methods in NTG-triggered attacks. We used resting state fMRI and seed-based connectivity analyses to examine the premonitory and headache phases of NTG-triggered migraine in a cohort of deeply phenotyped patients with migraine (*n* = 21) [33]. Each subject acted as their own control and was also imaged at the same time points during a triggered attack (baseline, premonitory, headache and recovery) and following placebo infusion in a randomised double-blind crossover design. We identified alterations in thalamocortical (bilateral thalami and precuneus and cuneus cortical regions) and pontolimbic (pons and limbic lobe) functional connectivity in the premonitory phase, and whilst the pontolimbic changes persisted through headache, other connectivity changes between the pons, medulla and cerebellar tonsils also emerged. We therefore postulated early functional reorganisation of sensory and limbic networks during the premonitory phase which could be mediating the clinical phenotype. A study by another group using NTG-triggered attacks and functional connectivity analyses in 5 subjects with 4 scans throughout the NTG-triggered migraine attack (baseline, prodromal, headache and recovery), provided supportive findings for temporal changes in thalamic function in the premonitory phase when NTG-triggered subjects were imaged during PS which were typical for them [35]. The authors found changes in connectivity between the right thalamus and insula, pons and cerebellum in the premonitory phase and a loss of synchronisation between the thalami and the salience network during this time, and these changes were also persistent during headache.

Most recently, we used 3D (three-dimensional) pCASL a non-invasive means of quantitatively measuring CBF in the same cohort of 21 NTG-triggered subjects we had previously imaged with resting state functional connectivity. We have shown that even in smaller numbers of more homogeneous patients, there were significant premonitory increases in CBF in anterior cingulate and frontal cortices, as well as in the caudate, lentiform, hippocampus and amygdala, and in the region of the hypothalamus on region of interest analysis [32]. These changes were only present in subjects not taking daily oral migraine prevention. This study has supported the role of the hypothalamus, but also other subcortical and cortical brain regions in mediating the premonitory phenotype. We have postulated the role of monoaminergic pathways in PS, including those involving dopamine via the hypothalamus, basal ganglia and ventral tegmentum, noradrenaline via the locus coeruleus and its projections and serotonin via the rostroventral medulla. These are all systems that have roles in trigeminal pain processing, as well as in the regulation of physiological mechanisms such as those involving sleep, mood and feeding, and are feasibly involved in the interactions of these factors with migraine, in terms of clinical symptoms and the perception of triggers. The same study also suggested that posterior hypoperfusion over the occipital cortices was present during the premonitory phase only in subjects with an underlying history of aura, irrespective of whether these aura symptoms were triggered with NTG or not. This raised interesting questions about a possible asymptomatic imaging correlate for aura, and supported the role of the occipital cortex hypoperfusion in aura mechanisms [67], although the outcomes of other imaging studies in this area have shown a combination of occipital hyper and hypoperfusion changes [68].

The studies performed during the triggered symptomatic premonitory phase of migraine are summarised in Table 2.

**Table 2:** Summary of the imaging studies conducted during the symptomatic premonitory phase of pharmacologically provoked migraine attacks

Study	Imaging modality	Subject cohort	Study design	Main findings
Maniyar *et al*., 2014 [30]	H_2_^15^0 PET	Migraine (*n* = 8)	Three scans per subject; baseline, premonitory and headache during NTG-triggered attacks	Increases in cerebral blood in hypothalamus, midbrain, dorsal pons and cortical areas during PS
Maniyar *et al*., 2014 [29]	H_2_^15^0 PET	Migraine with premonitory photophobia (*n* = 5) Migraine without premonitory photophobia (*n* = 5)	At least one premonitory scan per subject during NTG-triggered attacks	Greater activation of extrastriate visual cortex (BA18) during the premonitory phase in those with photophobia compared to those without relative to baseline
Maniyar *et al*., 2014 [31]	H_2_^15^0 PET	Migraine with premonitory nausea (*n* = 3) Migraine without premonitory nausea (*n* = 7)	Three scans per subject’ baseline, premonitory and headache during NTG-triggered attacks	Activation in rostral dorsal medulla and periaqueductal grey in the nausea group only
Karsan *et al*., 2020 [33]	Resting state fMRI with seed-based connectivity analysis	Migraine (*n* = 21)	Four scans per subject; baseline, premonitory, headache and resolution following NTG and placebo on two visits	Alterations in thalamus and cuneus and precuneus and pontolimbic functional connectivity in the premonitory phase Pontolimbic changes persisted through headache, with other changes involving pons, medulla and cerebellar tonsils
Martinelli *et al*., 2021 [35]	Resting state fMRI with seed-based correlation analysis and wavelet coherence analysis	Migraine (*n* = 5)	Four scans per subject; baseline, premonitory, headache and resolution during NTG-triggered attacks	Connectivity changes between right thalamus and insula, pons and cerebellum in the premonitory phase Loss of synchronisation between the thalami and the salience network
Karsan *et al*., 2023 [32]	3D pCASL	Migraine (*n* = 21)	Four scans per subject; baseline, premonitory, headache and resolution following NTG and placebo on two visits	Increases in cerebral blood flow in anterior cingulate and frontal cortices, caudate, lentiform, hippocampus and hypothalamus in those not on prevention Reduced occipital perfusion in those with underlying aura irrespective of whether aura was triggered or not

### Neurobiological and therapeutic insights

The pre-ictal or premonitory phase of migraine offers a unique opportunity to understand the possible mechanisms behind attack initiation. Despite advances in knowledge of the pathophysiology of migraine, the fundamental issue of how and where an attack starts anatomically remain unknown, and remain debated amongst headache researchers. Whilst dural activation, only near large vessels, produces headache phenotypically similar to migraine [69], headache is not the first symptom of the attack, and interictal and pre-ictal changes on functional brain imaging are evident in between attacks and leading up to an attack. Indeed, vasodilatation of extracerebral blood vessels is not a pre-requisite to developing migraine headache. The pre-headache brain changes, and the cyclical nature of some of them demonstrated throughout the migraine cycle, suggest that migraine is a primarily neural disorder of fluctuating brain changes in areas integral to pain processing, such as in the brainstem, but also in other areas important in sensory, limbic and homeostatic regulation. How these central changes contribute to or lead to headache remains unknown, but there is animal model evidence that even vasodilatory pharmacological migraine triggers such as NTG, have central effects on trigeminocervical complex and thalamus, and that triggering of an attack may be a central rather than peripheral phenomenon [2].

Central neuronal to peripheral dural nociceptor activation and an inflammatory cascade sensitising meningeal nociceptors, seems unlikely as a feasible theory of central initiation of migraine causing headache thereafter, especially in the context of an intact macrostructural blood–brain barrier in migraine [70–72]. It has become clear over time that many targeted and efficacious treatments for migraine abortion may not act solely peripherally and are present in albeit small concentrations in cerebrospinal fluid (CSF) in rodent models [73, 74], so there may be a central to peripheral mechanism in migraine pathophysiologically that is not yet understood. This has previously been suggested in migraine with aura, as a means of providing a link between cortical aura mechanisms and headache, and meningeal neuroinflammation and bridging vessels between brain and skull marrow have been suggested to be implicated [75, 76]. It is, however, possible that the central changes lead to a heightened perception of pain, and that usually innocuous stimuli, such as dural vessel pulsation, are perceived as abnormally painful during a migraine attack, due to preceding disturbance in brain sensory regulation. Moreover, a carefully studied case of a patient with attacks of migraine with and without aura has shown the same pattern of activation in the hypothalamus in the premonitory phase whether there is subsequent aura or not [77]. These data clearly suggest aura is not the trigger rather an epiphenomenon, occurring in parallel with other components of the attack [78]. The brain changes during PS or pre-ictally could also have a role in top-down modulation of trigeminal nociceptive processing and could mediate the interplay between different systems such as those regulating sleep and feeding, the interaction of perceived triggers and PS [79, 80], and sensory thresholding during the migraine cycle. Several monoaminergic and peptidergic systems are involved and some of these share roles in pain as well as in these other associated migraine symptoms. Whilst all the answers are not yet known, the imaging studies during the premonitory phase have certainly supported that the vascular and neuroinflammatory theories of migraine that have been posed in the literature cannot solely be responsible for the entire clinical phenotype of migraine, nor the brain changes demonstrated on imaging studies. Certainly, central neuronal mechanisms in complex and overlapping sensory and physiological systems are at play early in the attack.

The brain regions involved in PS that have emerged through the various imaging studies discussed involve complex networks that are involved in sensory, limbic and homeostatic regulation. These systems are closely linked with migraine, both symptomatically and in terms of trigger perception. For example, disrupted sleep (too much or too little) is a commonly reported migraine trigger, but sleep is often useful in aborting attacks and disrupted sleep and arousal changes are common PS. Another example is altered feeding; different foods such as sweets and chocolate, or skipping meals, are commonly reported migraine triggers, and food cravings for sweet things and anorexia are reported PS. These systems share anatomical substrates and neurochemical pathways with those involving trigeminovascular processing in migraine and provide avenues that may be explored moving forward with migraine therapeutics.

There has already been preclinical suggestion that targeting the neuropeptide Y (NPY) pathway in the hypothalamus, which has a role in appetite regulation, could hold therapeutic promise via the NPY Y1 receptor in migraine, as the pathway shares a role in trigeminovascular nociception in an animal model [59]. Modulating neuroendocrine pathways involved in feeding via leptin, insulin and glucose may also have a role in changing trigeminocervical neuronal responses in an animal model of migraine [58]. Furthering understanding of these systems, and the links between obesity and migraine and fluctuating blood sugars and migraine may provide therapeutic avenues through targeting systems involved in feeding, glucose and satiety regulation. The orexins (orexin A and B) are hypothalamic neurotransmitters also involved in feeding regulation, so these hypothalamic feeding networks may all be involved in migraine pathophysiology. Other areas that may be involved in these mechanisms include the ventral tegmentum [60], and here leptin may be involved in modulating dopaminergic signalling [81].

Preclinical work has suggested that the orexins may be involved in migraine via their roles in sleep and pain [82–84], and whilst a clinical trial of a dual orexin antagonist filorexant administered daily at night was not successful [85], more targeted therapeutics and different dosing regimens within this system may be a future therapeutic option for migraine. Arousal is also regulated via midbrain structures such as PAG and its projections to rostroventral medulla, as well as the locus coeruleus (LC) in the pons. This is a noradrenergic structure which is excited by orexins and has a possible role in migraine pathophysiology [66]. NPY has a contributory role in sleep regulation and sleep is closely linked to energy homeostasis, thus these hypothalamic feeding systems are closely interlinked with those controlling arousal, as well as those involved in stress (which also involves orexins, leptin and NPY) [2] and the motivation-cognition interaction [86].

Dopaminergic pathways have clearly been suggested to be involved in PS, given the dopaminergic nature to many symptoms [87], and the consistent hypothalamic involvement demonstrated on imaging studies. In addition, emerging evidence for involvement of dopaminergic networks via the nucleus accumbens, basal ganglia and ventral tegmentum, support this theory. Many anti-dopaminergic drugs have shown therapeutic efficacy in migraine, but these areas and dopaminergic pathways are involved in many other functions, that these drugs are not targeted for migraine and therefore do risk side effects.

The complex interplay between pain processing systems and other systems via shared anatomical brain regions and neurotransmitter systems, likely contributes to migraine being a genetically inherited trait of disordered sensory, limbic and homeostatic processing, which at times is triggered intrinsically or endogenously into an attack state. The role of perceived external triggers remains debatable [79, 80]. The attack involves brain regions and neurotransmitter systems that share pain processing with other physiological processes like sleep, feeding regulation, mood and cognition, and the interaction of these causes the heterogenous phenotype that patients experience including headache and non-headache symptoms during a migraine attack. The involvement of subcortical and cortical brain regions structurally and functionally and the early activation of hypothalamic, pontine and tegmental structures, as well as thalamic nuclei, leads to the disordered physiology which can precede headache and perhaps the misperception of some exogenous trigger factors. How and why this leads to headache is unknown, but thresholding may be at play, as well as top down modulation of trigeminovascular nociceptive processing via brainstem structures. The possible contribution of a peripheral dural component remains an area that warrants further attention.

Increasing understanding of the neurochemical systems within the brain regions that have emerged of interest from premonitory imaging may provide novel therapeutic targets. Targeting other non-pain systems identified within the overall neurobiology of migraine may provide the opportunity to treat headache as well as other disabling symptoms associated with migraine. Whilst there have been huge advances in migraine therapeutics in the last decade, additional treatment options will always be needed in a field where there are no biomarkers for treatment response prediction and a proportion of sufferers remain underserved by currently available therapies.

**Fig. 1:**
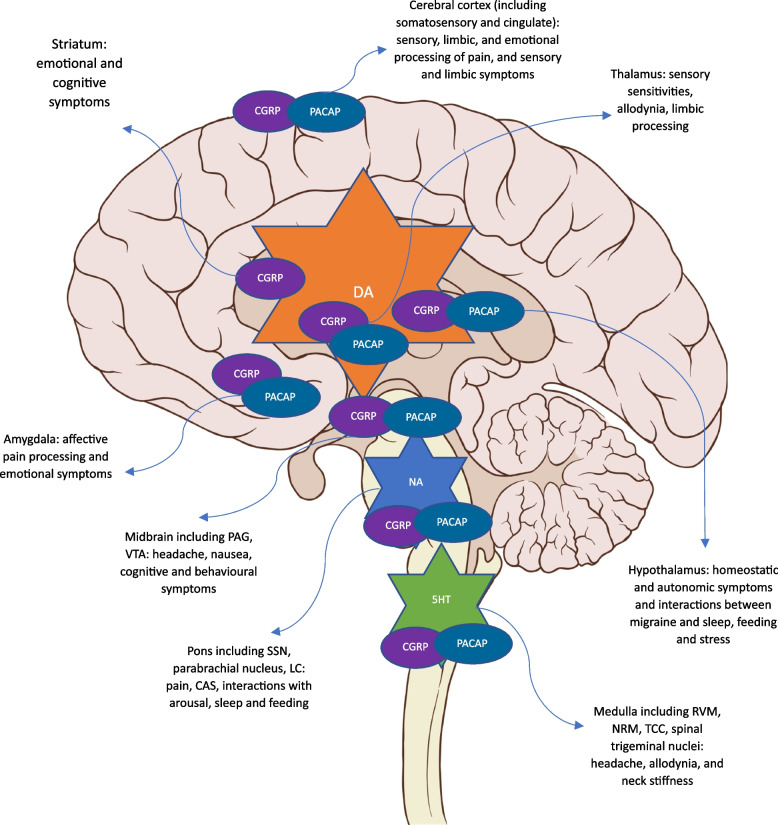
Brain regions involved in mediating PS and the proposed interaction between monoaminergic (stars) and peptidergic (ovals) pathways between these areas. DA; dopamine, NA; noradrenaline, 5HT; serotonin, CGRP; calcitonin gene-related peptide, PACAP; pituitary adenylate cyclase activating polypeptide; RVM; rostroventral medulla, NRM; nucleus raphe magnus, TCC; trigeminocervical complex, SSN; superior salivatory nucleus, LC; locus coeruleus, CAS; cranial autonomic symptoms, PAG; periacqueductal gray, VTA; ventral tegmental area. Free to use sagittal brain image https://upload.wikimedia.org/wikipedia/commons/a/a0/Brain_human_sagittal_section.svgby, Patrick J. Lynch, medical illustrator, CC BY 2.5 https://creativecommons.org/licenses/by/2.5, via Wikimedia Commons. The authors have annotated the figure for the purpose of this article

A proposed pathway of neuronal changes via several brain regions and neurochemical systems is shown in Fig. 1.

## Conclusions

In recent times, the evolution of human functional neuroimaging in migraine, as well as experimental migraine provocation, have provided the means to capture the earliest phase of the migraine attack with imaging methodologies. The use of non-invasive MR methodologies in particular with techniques such as resting state fMRI and ASL, which lack radiation exposure or radio-isotope use, have allowed the design of repeated measures imaging for such studies. The reliability of symptom triggering with phenotypic similarity across different episodes of pharmacological provocation have also supported these study designs [39]. Various imaging modalities of the pre-ictal or premonitory phase of migraine have contributed to a theory of disordered brain function early in the attack, involving areas involved in pain, sensory, limbic and homeostatic regulation such as the hypothalamus, basal ganglia, midbrain, pons, spinal trigeminal nuclei and limbic lobe. These areas are structurally and functionally connected via pathways involving dopamine, serotonin and noradrenaline, and also express peptides such as CGRP and PACAP which have demonstrated roles in migraine biology. This combination of monoaminergic and peptidergic brain dysfunction in areas shared between pain processing and other physiological systems is likely to contribute to the heterogeneous migraine phenotype, the association between symptoms and triggers, and the links to other symptoms and disorders such as those involving mood, sleep and cognition [88].

Interestingly, whilst NTG [38, 39, 41] and PACAP [40] can trigger PS when infused into patients with migraine (PACAP being another neuropeptide expressed in areas of interest in migraine, with a likely role in migraine pathophysiology [89–93] and future therapeutics [94–96]), CGRP has a lower tendency to do this [40]. However, treatments targeting the CGRP pathway have still shown effectiveness in preventing headache when taken during PS [15], and also efficacy in reducing the number of PS occurring with attacks when treatments are taken preventively [18]. This dissociation between triggering efficacy and targeted treatment efficacy remains poorly understood and certainly there is evidence CGRP may be involved in mediating some non-painful features of migraine such as photophobia [97] and cognitive dysfunction [98, 99], so there is a feasible way that this peptide could be involved in PS. PACAP has a role in mediating cranial autonomic symptoms (CAS) associated with migraine and cluster headache through expression in the sphenopalatine ganglion and cerebral vasculature [91, 94]. We have shown that CAS can be present before headache in the premonitory phase in experimentally-provoked attacks [39], so the activation of the parasympathetic reflex that causes CAS via the superior salivatory nucleus (SSN) in the pons does not require headache. This has also been demonstrated in cluster headache [100, 101], where these symptoms tend to be more prominent and are important in the diagnosis [6]. The early involvement of PACAP in the migraine attack is also likely, and whether targeted treatments towards this pathway show therapeutic efficacy in preventing PS remains to be discovered. The future of migraine therapeutics remains exciting as a result of the outcomes of functional imaging research of the migraine attack. The potential to treat before headache and prevent headache onset, and the ability of targeted therapies to prevent or treat non-headache symptoms of the attack are attractive concepts to patients and physicians alike. Further understanding of the neurobiology of PS and attack initiation, and the focussed targeting of these monoaminergic and peptidergic systems is likely to only increase the possible neurochemical targets in migraine treatment to offer more hope to those disabled by this common and heterogeneous condition.

## Data Availability

No new data was generated from this work and this manuscript reviews previous studies in the literature. All data discussed are cited appropriately and available within these references.

## Abbreviations

ASL: Arterial spin labelling
BOLD: Blood oxygen level dependant
CAS: Cranial autonomic symptoms
CBF: Cerebral blood flow
CGRP: Calcitonin gene-related peptide
CSD: Cortical spreading depression
CSF: Cerebrospinal fluid
DA: Dopamine
DTI: Diffusion tensor imaging
fMRI: Functional magnetic resonance imaging
LC: Locus coeruleus
NA: Noradrenaline
NPY: Neuropeptide Y
NRM: Nucleus raphe magnus
NTG: Nitroglycerin
PACAP: Pituitary adenylate cyclase activating polypeptide
PAG: Periacqueductal gray
pCASL: Pseudocontinuous arterial spin labelling
PET: Positron emission tomography
PS: Premonitory symptoms
RVM: Rostroventral medulla
SSN: Superior salivatory nucleus
TCC: Trigeminocervical complex
VTA: Ventral tegmental area
3D: Three dimensional
5HT: 5-Hydroxytriptamine or serotonin
